# Supplementary material for: Continuous plate subduction marked by the rise of alkali magmatism 2.1 billion years ago
Source: Nat Commun. 2019 Jul 30;10:3408. doi: 10.1038/s41467-019-11329-z (PMC6667441; doi:10.1038/s41467-019-11329-z)
Supplement: Supplementary file 1 — Supplementary Information [file 41467_2019_11329_MOESM1_ESM.pdf]

Supplementary Information for

Continuous plate subduction marked by the rise of alkali magmatism ~2.1 billion years ago

Liu et al.

Supplementary Figures

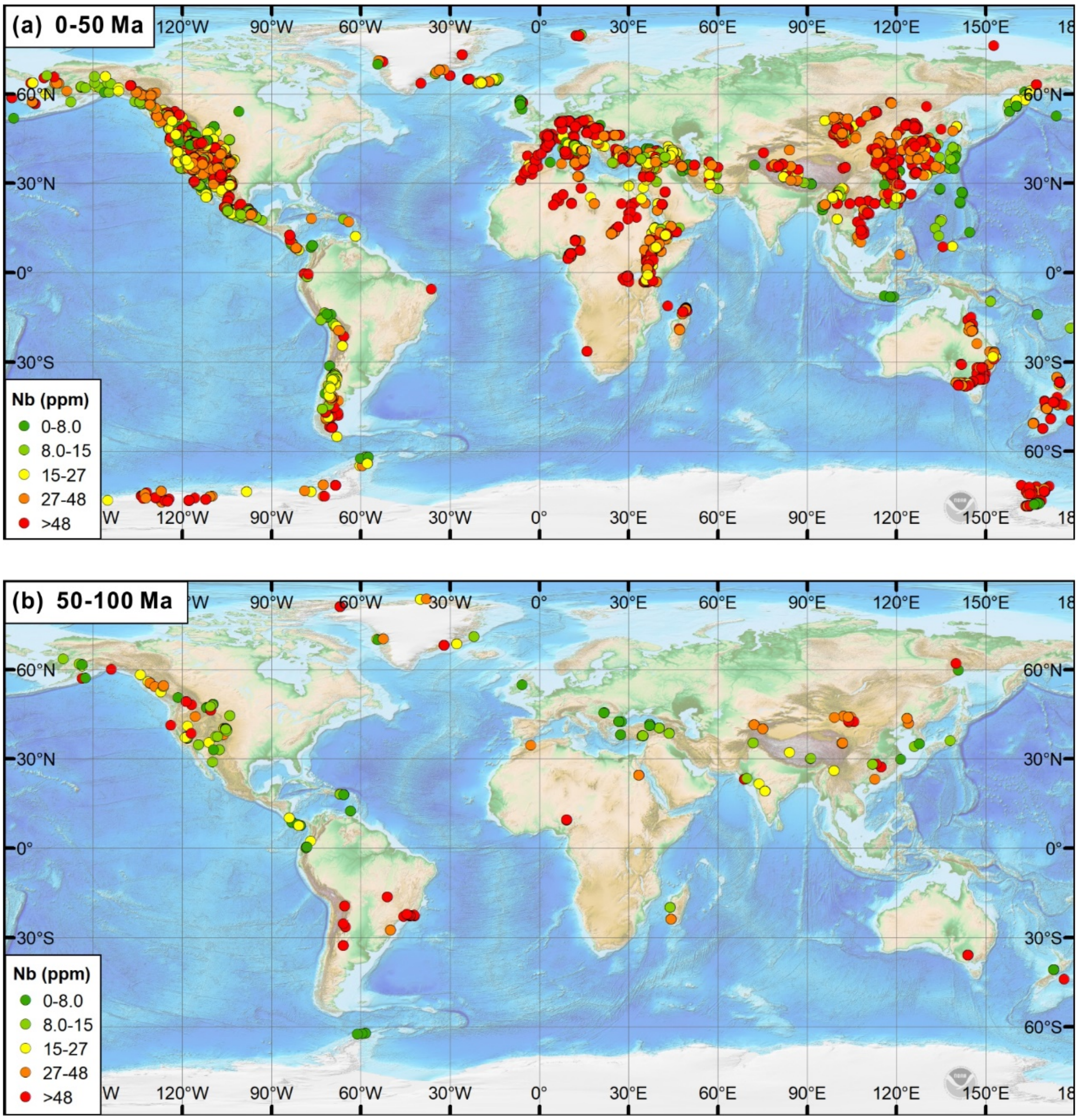

Supplementary Figure 1: Distribution of alkali basalts with ages of 0-100 Ma. (a) Distribution and Nb concentrations of alkali basalts with ages of 0-50 Ma. (b) Distribution and Nb concentrations of alkali basalts with ages of 50-100 Ma. The alkali basalts are selected using a Rittmann Index (σ) of an rock greater than 3.5[1]. $\sigma = (Na_2O + K_2O)^2/(SiO_2 - 43)$. In (a) many of the alkali basalts (high-Ti or low-Ti, refer to Farmer[2]) are located along the continental rifts and broad intracontinental regions, which formed by low-degree melting of upwelling asthenospheric mantle associated with the continental extension. Those alkali basalts contain high Nb concentrations. In (b) more alkali basalts are formed by melting of high-temperature mantle plumes, which have relatively lower Nb concentrations. Alkali basalts from continental rifts and other extensional settings contain higher Nb concentrations than those formed by mantle plumes. Background relief map is downloaded from NOAA website (https://www.ngdc.noaa.gov/)[3].

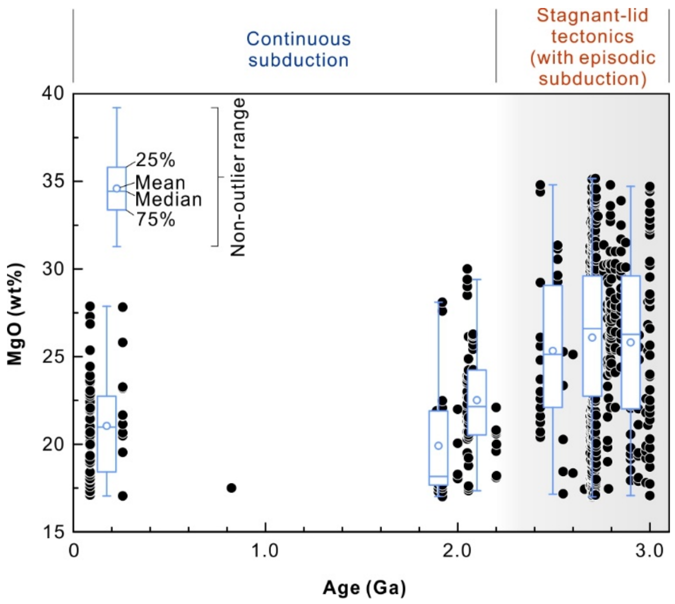

Supplementary Figure 2: Secular variation in MgO contents of komatiites (17-35 wt% MgO). The box plots show the mean, median, 25th-75th percentile and the non-outlier range (25th -75th percentile ± 1.5 * interquartile range) for the time windows (from left to right) for: 0-0.3, 1.8-2.0, 2.0-2.2, 2.4-2.6, 2.6-2.8, 2.8-3.0 Ga. The grey area shows the time range (>2.1 Ga) dominated by stagnant-lid tectonics with episodic subduction, while the white area is the time range (2.1-0 Ga) of continuous plate subduction. Data for komatiites are from Condie et al.[4]

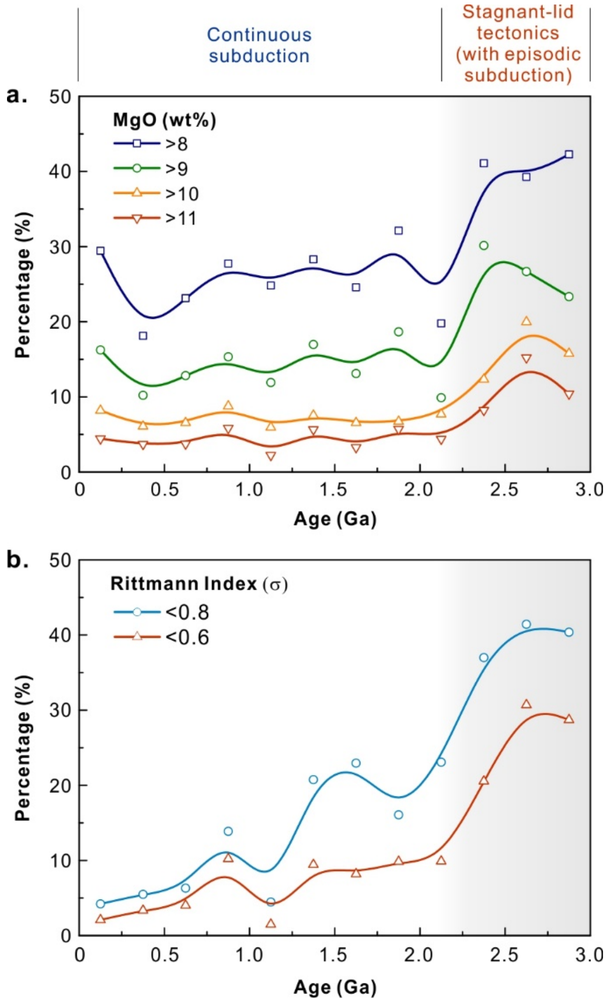

Supplementary Figure 3: Secular proportion of mafic rocks formed by high degree of mantle melting in relation to all mafic rocks. (a) Proportion (%) of mafic rocks with high MgO contents (>8, >9, >10, >11 wt% MgO) for every 0.25-Ga time bin from 3.0 to 0 Ga (see Supplementary Table 7). (b) Proportion (%) of mafic rocks with low Rittmann Index (σ<0.8, σ<0.6) for every 0.25-Ga time bin from 3.0 to 0 Ga (see Supplementary Table 8). The smooth curves are created using a cubic B-spline connection method given in OriginPro (v9.0). The grey area shows the time range (>2.1 Ga) dominated by stagnant-lid tectonics with episodic subduction, while the white area is the time range (2.1-0 Ga) of continuous plate subduction.

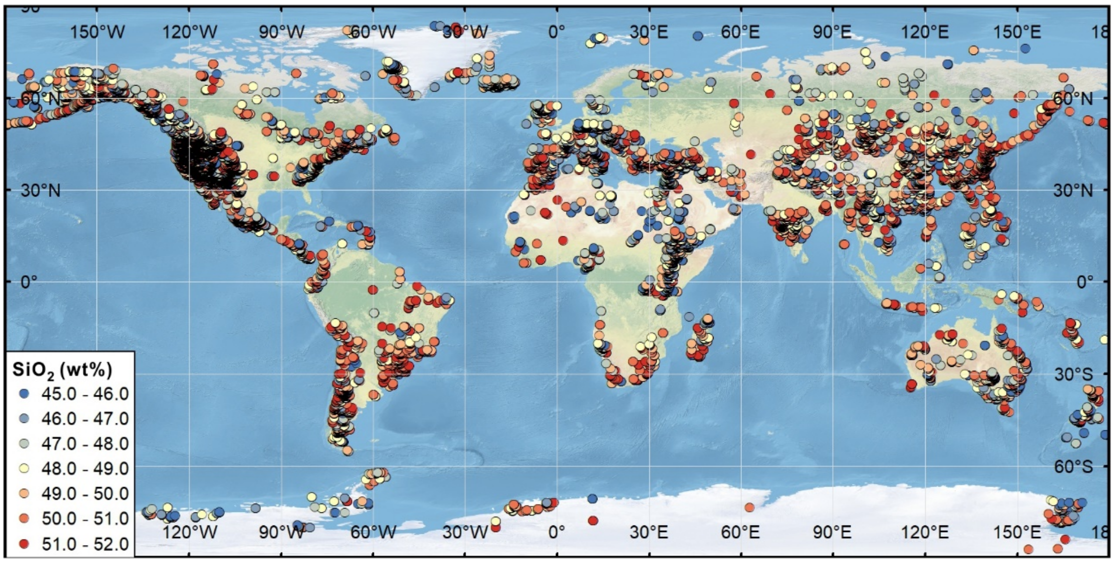

Supplementary Figure 4: Locations of mafic igneous rocks used in this study (55,107 samples). Samples on the oceanic floor have been removed from our dataset. All the data were downloaded from EarthChem database (http://www.earthchemportal.org/) with appropriate filtering. Background relief map is downloaded from NOAA website (https://www.ngdc.noaa.gov/)[3].

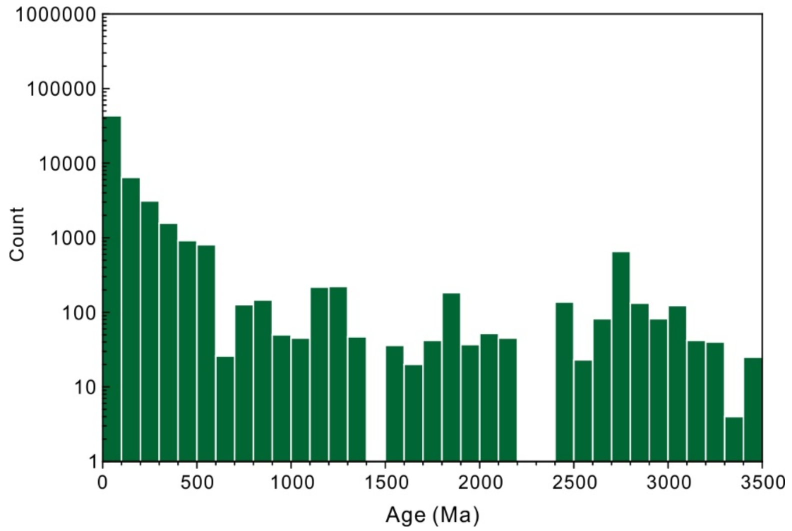

Supplementary Figure 5: Histograms showing the frequency of mafic rocks with 45-52 wt% SiO_2 (55,107 samples) over time. The y-axis scales are in logarithmic style.

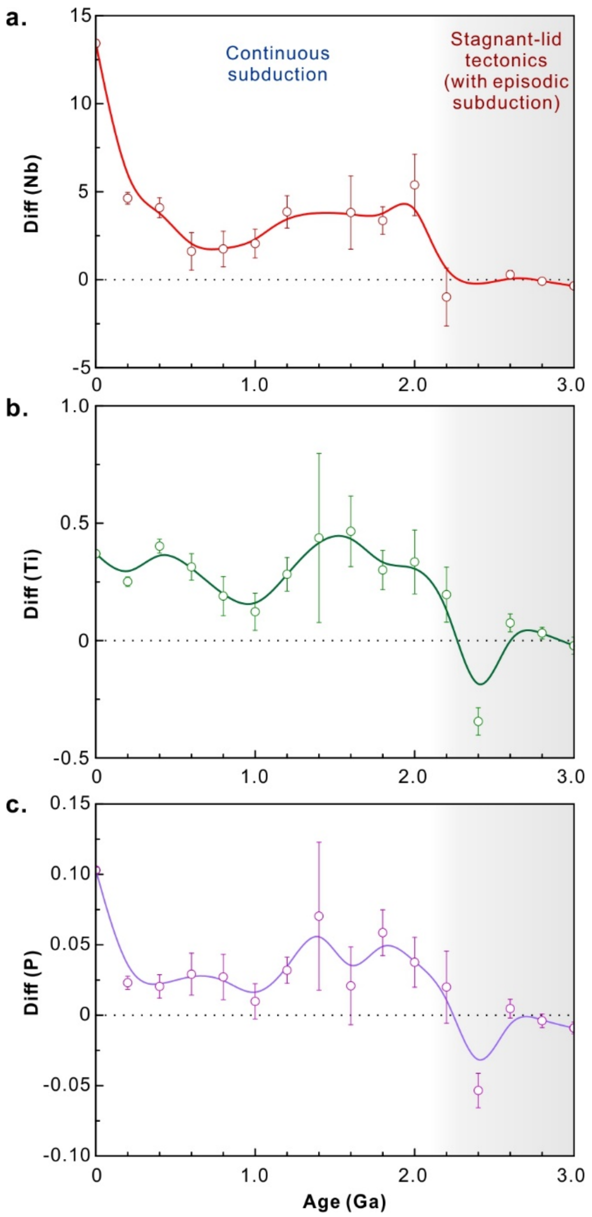

Supplementary Figure 6: Diff (HFSE) values over time. The Diff (HFSE) is a new geochemical proxy defined in this study to monitor the increase of alkali basaltic rocks. (a-c) Diff (Nb), Diff (Ti) and Diff (P), respectively, for every 0.2 Ga time bin from 3.0 to 0 Ga. Error bars represent the standard deviation (1 s.d.) of Diff (HFSE) estimated by the Monte Carlo method. The values at 0 Ga represent the Diff (HFSE) for 0.1-0 Ga. The smooth curves are created using a cubic B-spline connection method given in OriginPro (v9.0). The enhancement of Diff (Nb) and Diff (P) for the youngest 0.1 Ga time bin is caused by the abundantly sampled alkali basalts (both high-Ti and low Ti) from the continental rifts and volcanic cones in the broad continental areas for the most recent 50 Ma (Supplementary Figure 3). The grey area shows the time range (>2.1 Ga) dominated by stagnant-lid tectonics with episodic subduction, while the white area is the time range (2.1-0 Ga) of continuous plate subduction.

Supplementary Tables

Supplementary Table 1: Means and standard errors of Nb, TiO_2 and P_2O_5 vs SiO_2 concentrations for arc, intracontinental and all mafic rocks from East Asia.

Group	SiO_2 (wt%)	Nb (ppm)	2 s.e.m.	n	TiO_2 (wt%)	2 s.e.m.	n	P_2O_5 (wt%)	2 s.e.m.	n
All rocks	52	13.12	2.70	212	1.330	0.058	377	0.338	0.029	369
	51	15.08	2.13	404	1.346	0.044	722	0.315	0.020	704
	50	21.37	2.46	370	1.501	0.055	613	0.342	0.022	586
	49	28.96	2.23	351	1.838	0.066	498	0.450	0.025	474
	48	35.22	2.00	291	2.068	0.065	377	0.530	0.026	370
	47	40.87	2.77	176	2.199	0.073	225	0.620	0.033	225
	46	48.77	4.52	79	2.137	0.113	126	0.688	0.057	123
	45	51.00	6.32	27	1.947	0.192	50	0.701	0.104	47
Arc	52	6.53	1.04	175	1.180	0.048	322	0.290	0.026	316
	51	6.03	0.82	304	1.141	0.032	590	0.251	0.017	577
	50	5.60	1.19	205	1.110	0.035	420	0.215	0.016	398
	49	6.50	2.07	103	1.147	0.058	217	0.243	0.025	200
	48	6.97	4.06	28	1.131	0.101	82	0.250	0.043	78
	47	7.16	9.32	7	1.324	0.219	29	0.349	0.116	25
	46	10.88	15.61	4	1.466	0.227	35	0.376	0.129	30
	45	NaN	NaN	1	1.396	0.300	21	0.344	0.168	18
Intracontinent	52	43.34	8.82	38	2.259	0.092	53	0.622	0.092	53
	51	42.08	5.06	100	2.284	0.065	130	0.606	0.055	127
	50	41.67	3.68	164	2.348	0.058	192	0.611	0.039	188
	49	39.18	2.47	249	2.354	0.050	281	0.600	0.027	277
	48	38.66	2.05	264	2.319	0.048	295	0.605	0.024	295
	47	42.51	2.53	168	2.343	0.059	196	0.655	0.031	198
	46	51.43	4.09	74	2.404	0.098	94	0.772	0.049	92
	45	52.90	5.22	26	2.319	0.184	31	0.856	0.083	30

Supplementary Table 2: Means and standard errors of Nb, TiO_2 and P_2O_5 vs SiO_2 concentrations for arc, intracontinental and all mafic rocks from the southern Andes.

Group	SiO_2 (wt%)	Nb (ppm)	2 s.e.m.	n	TiO_2 (wt%)	2 s.e.m.	n	P_2O_5 (wt%)	2 s.e.m.	n
All rocks	52	4.73	2.45	45	1.244	0.098	79	0.252	0.038	78
	51	6.48	2.09	76	1.245	0.075	131	0.250	0.026	129
	50	14.38	2.67	81	1.464	0.081	120	0.335	0.028	117
	49	19.96	2.28	117	1.723	0.060	156	0.446	0.026	154
	48	19.78	1.84	137	1.780	0.054	175	0.447	0.021	176
	47	23.88	2.57	145	1.901	0.066	178	0.467	0.022	179
	46	28.86	3.57	105	1.976	0.091	126	0.533	0.027	128
	45	28.54	6.89	30	1.852	0.182	35	0.587	0.044	37
Arc	52	2.84	0.73	38	1.197	0.094	69	0.225	0.027	69
	51	2.94	0.52	59	1.153	0.069	107	0.222	0.020	107
	50	4.33	1.38	34	1.178	0.073	63	0.259	0.030	63
	49	6.96	3.52	17	1.379	0.103	36	0.328	0.039	37
	48	6.74	6.59	7	1.402	0.189	19	0.315	0.051	20
	47	3.64	1.09	3	1.470	0.273	13	0.326	0.075	13
	46	NaN	NaN	1	1.553	0.405	7	0.383	0.092	7
	45	NaN	NaN	1	0.987	0.749	2	0.339	0.170	2
Intracontinent	52	16.83	13.59	6	1.573	0.354	10	0.466	0.205	9
	51	18.64	6.46	17	1.691	0.202	23	0.387	0.097	22
	50	21.23	3.18	48	1.797	0.085	56	0.424	0.039	54
	49	22.76	2.54	101	1.822	0.060	119	0.483	0.028	117
	48	20.92	1.98	131	1.814	0.051	156	0.464	0.022	156
	47	24.30	2.55	142	1.926	0.068	166	0.478	0.023	166
	46	29.12	3.54	104	1.997	0.098	121	0.542	0.028	121
	45	29.44	7.07	29	1.901	0.194	35	0.600	0.042	35

Supplementary Table 3: Means and standard errors of Nb, TiO_2 and P_2O_5 vs SiO_2 concentrations for worldwide mafic rocks.

**SiO_2 (wt%)**	**Phanerozoic**			**Archean**		
	**Nb (ppm)**	**2 s.e.m.**	**n**	**Nb (ppm)**	**2 s.e.m.**	**n**
52	17.8	0.59	4688	3.7	0.57	173
51	17.9	0.40	9715	3.6	0.35	400
50	18.9	0.38	10631	3.4	0.27	449
49	21.5	0.39	11281	3.4	0.27	401
48	25.1	0.45	10831	3.4	0.45	298
47	30.5	0.56	8844	4.0	0.80	184
46	38.3	0.73	6594	4.7	1.38	91
45	42.9	1.16	2893	5.7	3.08	25

**SiO_2 (wt%)**	**Phanerozoic**			**Archean**		
	**TiO_2 (wt%)**	**2 s.e.m.**	**n**	**TiO_2 (wt%)**	**2 s.e.m.**	**n**
52	1.55	0.015	7869	0.89	0.055	199
51	1.61	0.012	16091	0.93	0.039	461
50	1.74	0.012	17265	0.96	0.037	514
49	1.82	0.012	18179	0.96	0.039	462
48	1.90	0.012	17673	0.96	0.048	343
47	2.05	0.014	14441	0.98	0.069	209
46	2.24	0.016	10382	1.04	0.108	107
45	2.32	0.025	4423	1.17	0.228	31

**SiO_2 (wt%)**	**Phanerozoic**			**Archean**		
	**P_2O_5 (wt%)**	**2 s.e.m.**	**n**	**P_2O_5 (wt%)**	**2 s.e.m.**	**n**
52	0.38	0.006	7620	0.11	0.014	185
51	0.39	0.004	15599	0.12	0.010	434
50	0.40	0.004	16702	0.11	0.011	488
49	0.41	0.004	17620	0.11	0.013	439
48	0.44	0.005	17213	0.11	0.013	327
47	0.49	0.005	14100	0.11	0.013	198
46	0.56	0.007	10130	0.11	0.017	99
45	0.60	0.011	4310	0.13	0.035	28

Supplementary Table 4: The Diff (Nb) of worldwide mafic rocks through Earth's history (3.0-0 Ga).

Age (Ma)	Low-silica mafic rocks			High-silica mafic rocks			Diff (Nb)	s.d.
	Mean Nb (ppm)	s.e.m.	n*	Mean Nb (ppm)	s.e.m.	n*		
3000	2.6	0.26	66	2.2	0.11	109	0.41	0.28
2900	2.1	0.16	99	2.1	0.09	135	-0.03	0.19
2800	2.3	0.12	127	2.5	0.09	189	-0.23	0.15
2700	2.8	0.09	299	3.0	0.07	497	-0.19	0.12
2600	3.0	0.11	315	3.1	0.07	497	-0.08	0.13
2500	3.2	0.12	281	3.3	0.08	453	-0.08	0.14
2400	3.5	0.13	246	3.7	0.10	468	-0.22	0.16
2300	3.6	0.15	218	3.8	0.11	428	-0.19	0.19
2200	3.8	0.20	150	4.0	0.14	294	-0.19	0.25
2100	4.9	0.70	20	5.9	0.43	71	-0.98	0.83
2000	9.7	1.00	29	7.8	0.50	85	1.95	1.12
1900	10.3	1.15	36	6.6	0.47	63	3.73	1.26
1800	8.8	0.64	82	6.0	0.26	142	2.83	0.68
1700	8.8	0.62	88	6.0	0.26	146	2.87	0.67
1600	8.8	0.62	83	5.7	0.25	152	3.12	0.67
1500	8.9	0.61	90	5.2	0.23	140	3.66	0.65
1400	8.5	0.67	81	5.2	0.22	139	3.35	0.70
1300	8.9	1.20	40	5.1	0.40	50	3.76	1.27
1200	9.9	1.29	39	6.1	0.58	51	3.81	1.40
1100	11.3	0.78	108	7.2	0.41	117	4.12	0.89
1000	10.8	0.75	99	7.1	0.44	101	3.70	0.86
900	10.5	0.72	107	7.0	0.41	103	3.50	0.82
800	9.2	0.55	155	6.7	0.34	149	2.48	0.65
700	9.1	0.56	150	6.5	0.34	132	2.66	0.65
600	8.4	0.48	151	6.6	0.36	121	1.83	0.60
500	12.0	0.64	351	10.3	0.54	257	1.64	0.84
400	13.3	0.46	633	10.0	0.40	507	3.35	0.60
300	14.8	0.36	1094	11.4	0.32	959	3.43	0.49
200	16.6	0.27	2183	12.0	0.23	1769	4.62	0.35
100	16.7	0.23	3436	12.3	0.15	3565	4.39	0.28
0	27.9	0.17	15311	16.0	0.10	13549	11.96	0.19

* Sample numbers in the 0.5-Ga moving window.

Supplementary Table 5: The Diff (Ti) of worldwide mafic rocks through Earth's history (3.0-0 Ga).

Age (Ma)	Low-silica mafic rocks			High-silica mafic rocks			Diff (Ti)	s.d.
	Mean TiO_2 (wt%)	s.e.m.	n*	Mean TiO_2 (wt%)	s.e.m.	n*		
3000	0.85	0.040	73	0.83	0.032	122	0.02	0.051
2900	0.82	0.029	105	0.83	0.027	147	0.00	0.039
2800	0.85	0.022	147	0.87	0.022	208	-0.01	0.031
2700	0.90	0.016	345	0.88	0.013	555	0.02	0.021
2600	0.92	0.017	368	0.90	0.012	570	0.02	0.021
2500	0.94	0.018	338	0.92	0.013	528	0.02	0.022
2400	0.97	0.020	302	0.96	0.013	549	0.01	0.024
2300	0.97	0.023	260	0.96	0.014	499	0.01	0.027
2200	1.02	0.031	177	1.00	0.018	342	0.03	0.035
2100	1.08	0.051	38	1.13	0.033	99	-0.06	0.061
2000	1.42	0.091	47	1.33	0.045	102	0.09	0.101
1900	1.68	0.102	44	1.37	0.056	75	0.31	0.116
1800	1.45	0.065	91	1.17	0.041	154	0.28	0.076
1700	1.43	0.059	102	1.17	0.040	159	0.26	0.071
1600	1.42	0.062	100	1.15	0.038	165	0.27	0.074
1500	1.48	0.061	99	1.07	0.036	143	0.41	0.070
1400	1.42	0.064	88	1.07	0.037	142	0.35	0.074
1300	1.74	0.094	54	1.22	0.053	54	0.52	0.109
1200	2.20	0.060	136	1.79	0.057	126	0.41	0.083
1100	1.99	0.050	217	1.68	0.043	199	0.31	0.066
1000	1.97	0.050	223	1.69	0.043	200	0.28	0.065
900	1.97	0.048	231	1.68	0.042	205	0.28	0.064
800	1.88	0.042	283	1.64	0.037	254	0.24	0.056
700	1.84	0.043	275	1.61	0.036	243	0.24	0.056
600	1.57	0.046	189	1.40	0.034	170	0.17	0.057
500	1.64	0.034	426	1.34	0.034	369	0.30	0.049
400	1.78	0.026	840	1.36	0.026	677	0.42	0.037
300	1.78	0.019	1424	1.40	0.017	1245	0.38	0.025
200	1.92	0.016	2791	1.47	0.013	2224	0.46	0.021
100	1.79	0.013	4584	1.51	0.010	4768	0.28	0.016
0	2.00	0.004	24681	1.65	0.004	22156	0.36	0.006

* Sample numbers in the 0.5-Ga moving window.

Supplementary Table 6: The Diff (P) of worldwide mafic rocks through Earth's history (3.0-0 Ga).

Age (Ma)	Low-silica mafic rocks			High-silica mafic rocks			Diff (P)	s.d.
	Mean P_2O_5 (wt%)	s.e.m.	n*	Mean P_2O_5 (wt%)	s.e.m.	n*		
3000	0.08	0.005	75	0.08	0.004	122	0.00	0.006
2900	0.07	0.003	109	0.08	0.003	150	-0.01	0.005
2800	0.07	0.003	147	0.09	0.003	209	-0.01	0.004
2700	0.08	0.003	338	0.09	0.002	535	-0.01	0.004
2600	0.09	0.003	347	0.09	0.002	546	0.00	0.004
2500	0.10	0.003	317	0.10	0.002	492	-0.01	0.004
2400	0.10	0.004	287	0.11	0.002	512	-0.01	0.004
2300	0.10	0.004	250	0.11	0.003	463	-0.01	0.005
2200	0.11	0.005	173	0.11	0.003	335	0.00	0.006
2100	0.11	0.011	40	0.13	0.007	100	-0.02	0.013
2000	0.14	0.015	48	0.15	0.007	100	0.00	0.017
1900	0.17	0.017	45	0.14	0.007	75	0.03	0.019
1800	0.18	0.012	94	0.14	0.006	161	0.04	0.013
1700	0.18	0.012	106	0.14	0.006	166	0.04	0.013
1600	0.18	0.012	101	0.14	0.006	168	0.04	0.013
1500	0.18	0.011	97	0.14	0.006	144	0.05	0.013
1400	0.19	0.013	86	0.13	0.006	142	0.05	0.014
1300	0.21	0.017	53	0.15	0.010	52	0.05	0.020
1200	0.21	0.008	139	0.18	0.007	123	0.03	0.011
1100	0.21	0.007	214	0.18	0.005	198	0.04	0.009
1000	0.21	0.007	222	0.18	0.005	199	0.03	0.009
900	0.21	0.007	231	0.18	0.005	205	0.03	0.008
800	0.21	0.006	285	0.19	0.005	252	0.02	0.008
700	0.20	0.006	276	0.18	0.005	249	0.03	0.008
600	0.19	0.007	189	0.17	0.006	172	0.02	0.010
500	0.27	0.008	420	0.23	0.008	350	0.04	0.012
400	0.28	0.006	827	0.24	0.006	648	0.04	0.008
300	0.32	0.005	1415	0.29	0.005	1200	0.02	0.007
200	0.32	0.003	2739	0.27	0.004	2167	0.04	0.005
100	0.31	0.003	4524	0.29	0.003	4661	0.02	0.004
0	0.46	0.001	24052	0.36	0.001	21478	0.09	0.002

* Sample numbers in the 0.5-Ga moving window.

Supplementary Table 7: The percentage of rocks with high MgO contents.

Age (Ma)	Number of all samples	Number of samples				Percentage (%)			
		MgO (wt%)							
		**>8**	**>9**	**>10**	**>11**	**>8**	**>9**	**>10**	**>11**
0-250	47895	14099	7781	3922	2127	29.4	16.2	8.2	4.4
250-500	3700	671	378	225	137	18.1	10.2	6.1	3.7
500-750	748	173	96	49	28	23.1	12.8	6.6	3.7
750-1000	137	38	21	12	8	27.7	15.3	8.8	5.8
1000-1250	403	100	48	24	9	24.8	11.9	6.0	2.2
1250-1500	53	15	9	4	3	28.3	17.0	7.5	5.7
1500-1750	61	15	8	4	2	24.6	13.1	6.6	3.3
1750-2000	193	62	36	13	11	32.1	18.7	6.7	5.7
2000-2250	91	18	9	7	4	19.8	9.9	7.7	4.4
2250-2500	73	30	22	9	6	41.1	30.1	12.3	8.2
2500-2750	611	240	163	122	93	39.3	26.7	20.0	15.2
2750-3000	317	134	74	50	33	42.3	23.3	15.8	10.4

Supplementary Table 8: The percentage of rocks with low Rittmann Index (σ).

Age (Ma)	Number of all samples	Number of samples			Percentage (%)		
		Rittmann Index (σ)					
		**< 1**	**< 0.8**	**< 0.6**	**< 1**	**< 0.8**	**< 0.6**
0-250	47686	3621	2008	995	7.6	4.2	2.1
250-500	3667	295	201	123	8.0	5.5	3.4
500-750	746	86	47	30	11.5	6.3	4.0
750-1000	137	21	19	14	15.3	13.9	10.2
1000-1250	403	33	18	6	8.2	4.5	1.5
1250-1500	53	15	11	5	28.3	20.8	9.4
1500-1750	61	18	14	5	29.5	23.0	8.2
1750-2000	193	43	31	19	22.3	16.1	9.8
2000-2250	91	27	21	9	29.7	23.1	9.9
2250-2500	73	33	27	15	45.2	37.0	20.5
2500-2750	606	304	251	186	50.2	41.4	30.7
2750-3000	317	172	128	91	54.3	40.4	28.7

Supplementary Table 9: The Diff (Nb) of worldwide mafic rocks for each 200-Ma time interval.

	Low-silica mafic rocks			High-silica mafic rocks				
**Age (Ma)**	**Mean Nb (ppm)**	**s.e.m.**	**n***	**Mean Nb (ppm)**	**s.e.m.**	**n***	**Diff (Nb)**	**s.d.**
3000	1.50	0.08	75	1.852	0.099	95	-0.35	0.13
2800	3.22	0.12	212	3.312	0.086	388	-0.09	0.15
2600	3.77	0.20	149	3.478	0.116	249	0.29	0.23
2400	-	-	1	7.066	0.684	47	-	-
2200	5.40	1.03	13	6.413	1.268	9	-0.98	1.64
2000	12.36	1.64	28	6.982	0.573	54	5.38	1.74
1800	8.41	0.75	60	5.056	0.223	109	3.36	0.78
1600	9.37	2.00	25	5.498	0.636	29	3.81	2.08
1400	10.07	1.80	8	-	-	2	-	-
1200	11.19	0.80	89	7.329	0.452	97	3.85	0.91
1000	9.24	0.68	87	7.172	0.467	73	2.06	0.82
800	7.47	0.86	57	5.724	0.531	49	1.75	1.01
600	13.53	0.83	283	11.915	0.680	204	1.61	1.07
400	15.38	0.42	810	11.283	0.377	753	4.09	0.57
200	17.35	0.29	2372	12.717	0.173	2611	4.63	0.34
0	30.81	0.19	12166	17.390	0.130	10107	13.42	0.23

Supplementary Table 10: The Diff (Ti) of worldwide mafic rocks for each 200-Ma time interval.

	Low-silica mafic rocks			High-silica mafic rocks				
**Age (Ma)**	**Mean TiO_2 (wt%)**	**s.e.m.**	**n***	**Mean TiO_2 (wt%)**	**s.e.m.**	**n***	**Diff (Ti)**	**s.d.**
3000	0.73	0.03	82	0.75	0.03	100	-0.02	0.04
2800	0.94	0.02	256	0.91	0.01	446	0.03	0.03
2600	1.04	0.03	168	0.96	0.02	288	0.08	0.04
2400	0.87	0.02	9	1.21	0.05	57	-0.34	0.06
2200	1.24	0.09	20	1.05	0.07	18	0.19	0.12
2000	1.75	0.12	33	1.41	0.06	63	0.33	0.14
1800	1.33	0.07	68	1.04	0.04	114	0.30	0.08
1600	1.68	0.14	25	1.22	0.07	29	0.47	0.15
1400	2.32	0.16	17	1.88	0.33	6	0.43	0.36
1200	1.99	0.05	197	1.71	0.05	181	0.28	0.07
1000	1.59	0.06	114	1.47	0.05	98	0.12	0.08
800	1.55	0.07	66	1.37	0.05	68	0.19	0.08
600	1.65	0.04	348	1.33	0.04	292	0.31	0.06
400	1.83	0.02	1066	1.42	0.02	934	0.40	0.03
200	1.79	0.02	3184	1.54	0.01	3554	0.25	0.02
0	2.05	0.00	20446	1.68	0.00	17589	0.37	0.01

Supplementary Table 11: The Diff (P) of worldwide mafic rocks for each 200-Ma time interval.

	Low-silica mafic rocks			High-silica mafic rocks				
**Age (Ma)**	**Mean P_2O_5 (wt%)**	**s.e.m.**	**n***	**Mean P_2O_5 (wt%)**	**s.e.m.**	**n***	**Diff (P)**	**s.d.**
3000	0.055	0.002	83	0.064	0.003	103	-0.009	0.004
2800	0.098	0.004	238	0.101	0.003	410	-0.004	0.005
2600	0.110	0.006	162	0.105	0.003	279	0.005	0.007
2400	0.086	0.008	10	0.140	0.010	55	-0.053	0.012
2200	0.124	0.022	20	0.104	0.013	18	0.020	0.026
2000	0.171	0.016	33	0.133	0.007	64	0.038	0.018
1800	0.191	0.015	68	0.133	0.007	116	0.059	0.016
1600	0.159	0.023	22	0.138	0.016	28	0.021	0.028
1400	0.246	0.025	17	0.176	0.047	5	0.070	0.052
1200	0.211	0.007	198	0.179	0.006	183	0.032	0.009
1000	0.185	0.009	114	0.175	0.008	99	0.010	0.012
800	0.193	0.011	66	0.166	0.011	71	0.027	0.016
600	0.288	0.011	343	0.259	0.010	263	0.029	0.015
400	0.329	0.005	1049	0.308	0.006	908	0.020	0.008
200	0.309	0.003	3111	0.286	0.003	3453	0.023	0.005
0	0.487	0.002	19893	0.384	0.002	17158	0.103	0.002

Supplementary References

1 Yang, X.-M. Using the Rittmann Serial Index to define the alkalinity of igneous rocks. *Neues Jahrbuch für Mineralogie - Abhandlungen* **184**, 95-103, doi:10.1127/0077-7757/2007/0082 (2007).

2 Farmer, G. L. in *Treatise on Geochemistry (Second Edition)* (eds Heinrich D. Holland & Karl K. Turekian) 75-110 (Elsevier, 2014).

3 Amante, C. & Eakins, B. W. ETOPO1 arc-minute global relief model: procedures, data sources and analysis. NOAA Technical Memorandum. Report No. NESDIS NGDC-24, 1-19 (National Geophysical Data Center, NOAA, Boulder, Colorado, 2009).

4 Condie, K. C., Aster, R. C. & van Hunen, J. A great thermal divergence in the mantle beginning 2.5 Ga: Geochemical constraints from greenstone basalts and komatiites. *Geosci Front* **7**, 543-553, doi:10.1016/j.gsf.2016.01.006 (2016).
