# Supplementary material for: Continuous plate subduction marked by the rise of alkali magmatism 2.1 billion years ago
Source: Nat Commun. 2019 Jul 30;10:3408. doi: 10.1038/s41467-019-11329-z (PMC6667441; doi:10.1038/s41467-019-11329-z)
Supplement: Supplementary file 3 — Description of Additional Supplementary Files [file 41467_2019_11329_MOESM3_ESM.docx]

**Description of Supplementary Files**

**File Name:** **Supplementary Data 1**

**Description:** This Excel file contains the compiled dataset used in this study, including geochemical data of 55,107 igneous rocks.
